# Potential Influence of Climate Change on Vector-Borne and Zoonotic Diseases: A Review and Proposed Research Plan

**DOI:** 10.1289/ehp.0901389

**Published:** 2010-06-24

**Authors:** James N. Mills, Kenneth L. Gage, Ali S. Khan

**Affiliations:** National Center for Emerging and Zoonotic Infectious Diseases, Centers for Disease Control and Prevention, Atlanta, Georgia, USA

**Keywords:** anthropogenic disturbance, climate change, infectious diseases, reservoir, vector, vector-borne disease, wildlife, zoonotic disease

## Abstract

**Background:**

Because of complex interactions of climate variables at the levels of the pathogen, vector, and host, the potential influence of climate change on vector-borne and zoonotic diseases (VBZDs) is poorly understood and difficult to predict. Climate effects on the nonvector-borne zoonotic diseases are especially obscure and have received scant treatment.

**Objective:**

We described known and potential effects of climate change on VBZDs and proposed specific studies to increase our understanding of these effects. The nonvector-borne zoonotic diseases have received scant treatment and are emphasized in this paper.

**Data sources and synthesis:**

We used a review of the existing literature and extrapolations from observations of short-term climate variation to suggest potential impacts of climate change on VBZDs. Using public health priorities on climate change, published by the Centers for Disease Control and Prevention, we developed six specific goals for increasing understanding of the interaction between climate and VBZDs and for improving capacity for predicting climate change effects on incidence and distribution of VBZDs.

**Conclusions:**

Climate change may affect the incidence of VBZDs through its effect on four principal characteristics of host and vector populations that relate to pathogen transmission to humans: geographic distribution, population density, prevalence of infection by zoonotic pathogens, and the pathogen load in individual hosts and vectors. These mechanisms may interact with each other and with other factors such as anthropogenic disturbance to produce varying effects on pathogen transmission within host and vector populations and to humans. Because climate change effects on most VBZDs act through wildlife hosts and vectors, understanding these effects will require multidisciplinary teams to conduct and interpret ecosystem-based studies of VBZD pathogens in host and vector populations and to identify the hosts, vectors, and pathogens with the greatest potential to affect human populations under climate change scenarios.

The concept that weather and climate are linked to the incidence of infectious diseases in humans has been recognized since the time of Hippocrates (National Research Council 2001). Understanding the link between climate and disease has increasing urgency. According to the Intergovernmental Panel on Climate Change (IPCC 2007), warming of the earth’s climate is “unequivocal.” Global changes already documented include increased global surface temperature, rising sea level, decreased arctic and alpine snow and ice, and evidence of plants and animals responding to these changes by moving to higher elevations or closer to the poles. Precipitation has increased in some parts of the world while decreasing in others. These changes are predicted to continue into the foreseeable future (IPCC 2007).

Climate change is predicted to have a variety of impacts on human health, many of which have been extensively reviewed (Confalonieri et al. 2007; Ebi et al. 2006, 2008; Frumkin et al. 2008; Patz and Olson 2006). Some of these effects are easily intuited: increasing temperatures will likely increase the incidence of heat-related mortality (and decrease the risk mortality from cold exposure); increased drought will result in increased food and water shortages, whereas more frequent and more severe heavy precipitation events will increase the incidence of flood-related injuries and deaths, water-and food-borne illness, and other infectious, respiratory, and skin diseases.

Another class of potential impacts on human health often cited as a consequence of climate change is less direct, less intuitive, and less predictable: Those caused by infectious diseases acquired by humans from invertebrate and vertebrate animals—the vector-borne and zoonotic diseases (VBZDs). Zoonotic diseases are those caused by pathogens that are transmitted between vertebrate animals and humans. Vector-borne diseases are those for which the pathogen is transmitted to or among humans by an arthropod vector. Zoonotic diseases may be vector borne (transmitted to humans from a vertebrate reservoir by an arthropod vector such as Lyme disease) or nonvector borne [transmitted to humans by direct or indirect contact with the vertebrate host without the participation of an arthropod vector such as hantavirus pulmonary syndrome (HPS)]. Vector-borne diseases, in turn, may be zoonotic, as in the Lyme disease example, or nonzoonotic (transmitted by an arthropod vector from human to human without the involvement of a nonhuman reservoir such as malaria). We have used the inclusive term “VBZD” to include all three possible categories [for additional definitions, examples, and background information, see Table 1 and Supplemental Material (doi:10.1289/ehp.0901389)]. Making predictions concerning the effects of VBZDs adds additional complexity to forecasts. Although understanding the transmission of any infectious pathogen requires knowledge of the interactions between the pathogen and humans, in the case of VBZDs we must also understand the interactions of the pathogen with the arthropod vector and with the vertebrate host. Thus, a complete understanding of VBZDs requires in-depth knowledge of the ecology and natural history of the host or vector (Gage et al. 2008; Mills and Childs 1998).

In this article, we have proposed four primary mechanisms by which climate change is likely to affect pathogen, host, and vector populations; summarized current evidence that these mechanisms affect host and vector populations; and assessed how these effects are likely to influence human health. We also have attempted to identify important gaps in our knowledge and have suggested studies that are needed to fill those gaps and provide reliable information about the ultimate effects of climate change on VBZDs.

## Expected Changes

If climate change is to affect the frequency of VBZDs through acting on nonhuman hosts, vectors, and pathogens, it is likely to act by one (or a combination) of four primary mechanisms: *a*) range shifts in host or vector distribution that bring these hosts and vectors into contact with new human populations; *b*) changes in the population density of the host or vector that would result in increased or decreased frequency of contact with humans or with other hosts and vectors; *c*) changes in the prevalence of infection by the pathogen in the host or vector population that would increase or decrease the frequency of human (or other host or vector) contact with an infected host or vector; and *d*) changes in pathogen load brought about by changes in rates of pathogen reproduction, replication, or development in hosts or vectors that would affect the likelihood that a human (or other host or vector) contact would result in pathogen transmission.

### The evidence: what do existing studies portend? Range shifts

Geographic changes in the distributions of wildlife species in response to climate change are difficult to document because of the absence of current and historic data on distributions of many species and populations (Jannett et al. 2007; Thomas et al. 2006). Nevertheless, range shifts have been observed for a variety of taxa, including important mammalian hosts and arthropod vectors. These range shifts have generally been poleward and upward (toward higher elevations) (Hickling et al. 2006; Rosenzweig et al. 2007), and they have resulted in overall expansions, contractions, or no change in the total area occupied by a population or species. Altitudinal and latitudinal range shifts have occurred for *Ixodes ricinus*, the vector of the agents of Lyme disease and tick-borne encephalitis (TBE) in Europe (reviewed by Gage et al. 2008), and these shifts are associated with (“association” as used throughout this review is not proof of causation) new foci of disease and an increase in incidence of TBE (Kerr 2001; Lindgren and Gustafson 2001). Northerly range shifts also have been observed for *Ixodes scapularis*, a vector of Lyme disease, human granulocytic anaplasmosis, and babesiosis in North America. Models indicate that this species will extend its range farther into Canada while contracting its southern range (Brownstein et al. 2005; Ogden et al. 2006, 2010).

The hispid cotton rat (*Sigmodon hispidus*), which occurs in the southern United States, is the host of Black Creek Canal hantavirus (Table 1), a known cause of HPS (Rollin et al. 1995) as well as Muleshoe hantavirus (Rawlings et al. 1996), and Tamiami arenavirus (Calisher et al. 1970). The distribution of the hispid cotton rat is climate limited, and precise temperature minima for the species’ distributional limits have been described (Mohlhenrich 1961). Nevertheless, that distribution has undergone a northward expansion (Cameron and Spencer 1981; Schmidly 2002) in the last few decades, and a new altitudinal record was described for hispid cotton rats in New Mexico (Dunnum 2002). The white-footed deer mouse (*Peromyscus leucopus*), a host for the agents of Lyme disease, human granulocytic anaplasmosis, babesiosis, and HPS, has extended its range northward in Wisconsin (Long 1996) and Minnesota (Jannett et al. 2007). Hispid cotton rats and white-footed deer mice are common species in the United States and have been studied by generations of mammalogists. Little or no baseline data are available for the huge number of rodents in the tropics, the area where hosts (especially rodents and bats), vectors, and pathogens have their greatest diversity, yet they are the least studied [for a discussion of tropical disease diversity, rodents, and bats, see Supplemental Material (doi:10.1289/ehp.0901389)]. Known and unknown tropical host and pathogen systems are likely to shift their ranges closer to subtropical and temperate population centers as climate changes at higher latitudes.

**Figure 1:**
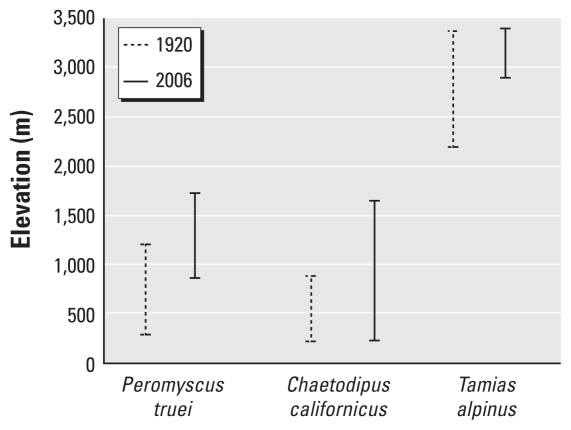
Elevational distribution of the piñon deer mouse (*Peromyscus truei*), the California pocket mouse (*Chaetodipus californicus*), and the alpine chipmunk (*Tamias alpinus*) in Yosemite National Park, California, 1920 and 2006 (Moritz et al. 2008).

Altitudinal temperature gradients are about 1,000 times steeper than latitudinal gradients (Colwell et al. 2008), which makes altitudinal transects more practical models for studying the effects of temperature change on plant and animal distributions. A recent range survey of small mammals in Yosemite National Park, California (USA), serves as a model (Moritz et al. 2008). Compared with similar surveys conducted approximately 100 years earlier (when minimum temperatures were 3°C lower), half of the 28 species monitored showed large (average = 500 m) upward changes in elevational limits (Figure 1). Low-altitude species shifted both their lower and upper range limits upslope (e.g., the piñon deer mouse, *Peromyscus truei*) or expanded their upper range upslope while maintaining a stable lower range limit (e.g., the California pocket mouse, *Chaetodipus californicus*). High-altitude species such as the alpine chipmunk (*Tamias alpinus*) showed overall range contractions because of upslope shifts in their lower limits. A continuation of this pattern suggests the ultimate extinction of some high-altitude species.

The latitudinal temperature gradient in the tropics is relatively shallow. Not only does this make latitudinal studies more difficult, but it portends relatively higher extinction rates for midtropical species of restricted geographic range and limited dispersal capabilities. Elevational shifts provide the only published data for range shifts in the tropics, where upslope range shifts have been demonstrated for plants and vertebrate animals (Colwell et al. 2008; Pounds et al. 2005). Unlike most temperate areas, in the lowland tropics there is no community of species adapted to even hotter areas available to replace those species that shift their ranges upslope or poleward. Thus, the species diversity of lowland tropical areas is expected to decrease as global temperatures rise (Colwell et al. 2008). However, extinctions are likely to be selective. Specialist species with relatively narrow niche breadth (especially in terms of physiological tolerances, habitat, and diet requirements) are most likely to suffer, whereas more adaptable generalist species may thrive, with potentially important consequences for pathogen transmission (Mills 2006).

Range shifts may not be restricted to latitudinal and elevational dimensions. Several host and vector species are restricted to specific habitat types. The frequency of occurrence of hosts, vectors, or pathogens in these habitat types helps to define relative risk of disease to humans associated with these habitats (Mills and Childs 1998). Effects of climate change on the species composition of plant and animal communities are poorly understood. Range shifts in response to climate change are unlikely to affect entire communities or assemblages in unison (Root and Schneider 2002). Species will migrate at different rates and according to their own motility, tolerances, and physiological constraints. Thus, species assemblages are likely to change: Some species in new areas will experience increased population densities and competitive release, allowing colonization of habitats from which they were formerly excluded, whereas others may experience increased pressures and decreased population densities as new competitors or predators move into their ranges or they are unable to migrate as climate change leaves them stranded on shrinking islands of suitable habitat. This concept was illustrated by several high-altitude rodent and insectivore species in Yosemite National Park that are now restricted to only a portion of their former range (Moritz et al. 2008) (Figure 1).

### Changes in population density

Climate change has been associated with conditions that alter the carrying capacity of ecosystems for some plant or animal populations (Gage et al. 2008; Sutherst 2004). Such changes can result in dramatic increases, or sometimes decreases, in host or vector population densities. Examples from vector-borne diseases are numerous. Populations of *Aedes* mosquitoes (vector for the agent of Rift Valley fever in East Africa) (Table 1) increase dramatically after periods of high rainfall associated with El Niño Southern Oscillation (ENSO) events (Anyamba et al. 2001; Linthicum et al. 1999). Increases in mosquitoes and mosquito-borne malaria were associated with ENSO events in South America (Barrera et al. 1999; Bouma and Dye 1997; Bouma et al. 1997) and southern Africa (Mabaso et al. 2007). Increasing temperatures also can decrease the development time of mosquitoes allowing greater population densities to be reached (Gage et al. 2008). Increased incidence of malaria was linked to precipitation and higher temperatures in Kenya (Githeko and Ndegwa 2001) and Madagascar (Bouma 2003). Conversely, reduced rainfall in Senegal and Niger was associated with decreases in malaria (Julvez et al. 1997; Mouchet et al. 1996), presumably resulting from decreased mosquito reproduction.

The effects of environmental extremes on vertebrate host populations can be pronounced. High summer temperatures have resulted in increased mortality of hosts, such as bats in tropical or subtropical areas (Welbergen et al. 2008) or increased overwinter survivorship of species in temperate areas (Straile and Stenseth 2007). After an ENSO event in 1997, investigators recorded increased rainfall, improved quality of vegetation, and increased population density of North American deer mice (*Peromyscus maniculatus*) in the southwestern United States. These events preceded increased numbers of HPS cases in the area over 3 years (Yates et al. 2002). Increased rainfall was also associated with outbreaks of HPS in Paraguay (Williams et al. 1997) and Panama (Ruedas et al. 2004). Similarly, human plague (a flea-borne zoonotic disease caused by the bacterium *Yersinia pestis*; Table 1) in the western United States was influenced by the effects of above-normal temperatures and status of the Pacific Decadal Oscillation (PDO). The PDO resulted in precipitation increases (Ari et al. 2008) that were believed to enhance small-mammal food resources and flea survival (Enscore et al. 2002; Parmenter et al. 1999). Although less attention has been paid to decreases in host populations, lower rainfall is accompanied by decreasing populations of North American deer mice in the southwestern United States (Yates et al. 2002), whereas increasing rainfall during cold periods was associated with abrupt population declines in some ecosystems (Calisher et al. 2005).

### Changes in prevalence of infection in host or vector populations

The increases in host or vector populations discussed above can lead to increased frequency of contact between hosts or between hosts and vectors (density-dependent transmission) (Begon et al. 2002) and increased prevalence of infection in host or vector populations. The increased North American deer mouse population density in the southwestern United States that followed the 1997 ENSO was followed, a year later, by increased prevalence of hantavirus infection in North American deer mouse populations [Centers for Disease Control and Prevention (CDC) 1999; Yates et al. 2002]. This delayed density-dependent increase in infection in host populations may be a general phenomenon affecting horizontally transmitted pathogens in wildlife hosts in strongly seasonal climates (Madhav et al. 2007; Mills et al. 1999). Similarly, as rodent populations increase above certain thresholds, the likelihood of plague epizootics also may increase (Davis et al. 2004). Precipitation and temperature influence the appearance of plague epizootics among Asian gerbils (Stenseth et al. 2006) and epizootics among prairie dogs in Colorado were linked to ENSO events (Stapp et al. 2004).

Prevalence of infection for other VBZDs might be increased by other mechanisms besides density-dependent transmission. Environmental changes that lead to increased stress in hosts can decrease host immune response, resulting in increased probability of infection as well as higher pathogen loads and increased pathogen shedding by a variety of host species (Nelson et al. 1995), including hosts for hantaviruses (Demas and Nelson 1998; Klein et al. 2004; Lehmer et al. 2007) and arenaviruses (Barnard et al. 1996).

### Changes in pathogen load

Temperatures can have profound effects on the development of pathogens and pathogen loads in arthropod vectors (Gage et al. 2008; Sutherst 2004). Malaria parasites develop in mosquito vectors only within certain temperature ranges (Patz and Olson 2006). Similarly, *Y. pestis*, the etiological agent of plague, develops and expresses biofilm efficiently only at temperatures < 28°C. Biofilm facilitates *Y. pestis* transmission by causing infected fleas to increase feeding attempts and regurgitate *Y. pestis* back into host animals during feeding (Gage and Kosoy 2005; Jarrett et al. 2004). Survival of *Y. pestis* also is influenced by temperature with many fleas clearing infection as temperatures increase above 28°C (Gage and Kosoy 2005; Hinnebusch et al. 1998).

Recurrence of viral replication (recrudescence) under stress, including heat stress, has been documented for human viral diseases (Halford et al. 1996; Mehta et al. 2004) and is likely to occur within zoonotic host populations also. Replication and shedding of Sin Nombre virus (Table 1) are detected intermittently in infected North American deer mice (Botten et al. 2003; Kuenzi et al. 2005). Although mechanisms for the reactivation of viral replication are poorly understood, stress-related immunosuppression has been suggested (Botten et al. 2002; Kuenzi et al. 2005). The relationship between stress and pathogen transmission, replication, persistence, and shedding in hosts and vectors is poorly studied. A clearer understanding of this relationship would lead to more accurate predictions of the effects of climate change on VBZD risk to humans.

### Interactions among climate variables

The amplitude and even the direction of effects of climate on host and vector populations are locally variable and depend upon interactions with other variables (Mills 2005). Contrary to what was observed in New Mexico, the 1997 ENSO did not lead to a net increase in North American deer mouse populations at a high-altitude site in Colorado. Increases in abundance of North American deer mice in Colorado from 1995 to 2000 were associated with increased rainfall during warm periods, but populations crashed after increased rainfall during cold periods (Calisher et al. 2005). Similarly, the combined effects of temperature and humidity affect the behavior, survival, and reproduction of many vector species. Survival of ticks and fleas are negatively affected by the combination of hot and dry conditions (Gage and Kosoy 2005; Randolph 2004). The same conditions limit the ability of ticks to quest for hosts in exposed environments.

These examples indicate that host responses to climate change are multifactorial and their quantitative prediction must consider multiple variables simultaneously, including temperature, precipitation, altitude, and location. The separation and independent measurement of these interacting effects have been identified as a research priority (Harvell et al. 2002). Nevertheless, because these factors contribute to overall effects interactively and often operate synergistically, predictions using single variables must be subject to cautious interpretation.

### Interactions of climate change with anthropogenic factors

Just as the interaction effects between various aspects of climate change (e.g., temperature and precipitation) must be considered to accurately predict the influence of climate on VBZDs, so too must the interactions between climate change and other anthropogenic and natural factors. Human activities facilitate host or vector range shifts by transporting hosts or vectors to new geographic areas (Benedict et al. 2007), including new continents, as was the case with vectors of plague (Parmenter et al. 1999) and West Nile virus (Komar 2003) and hosts of monkeypox in the United States (Likos et al. 2005). Human migrations in response to climate change may make them more vulnerable to some VBZDs. For example, increases in leishmaniasis were associated with drought-related clustering of humans near water supplies where sand fly vectors were concentrated (Thompson et al. 2002). Human cultural and behavioral differences will alter the end effect of all of the mechanisms discussed above. Although *Aedes aegypti*, the primary vector of Dengue virus, is likely to achieve northward range expansions in the United States as winter temperatures become warmer, this may not result in dengue epidemics in the United States. In southern Texas, where *Ae. aegypti* is already established, dengue has remained rare despite epidemics on the Mexican side of the border. This is thought to be due to human-related factors, such as the greater use of air conditioning in the United States, which decreases exposure to mosquitoes (Reiter et al. 2003). Finally, the degree of human intervention to prevent or mitigate adverse effects of climate change on health will affect the eventual outcomes of all of the mechanisms discussed above. This is a large field of study (Confalonieri et al. 2007) but is outside the scope of our discussion.

Anthropogenic disturbance of ecosystems will modify the response of many species to climate change. As mentioned, the distributional range of many wildlife species and vectors is predicted to shift poleward and toward higher altitudes as the climate warms, perhaps bringing hosts, vectors, and diseases currently restricted to the tropics within the range of temperate population centers. Similar species migrations occurred at the transition from the last ice age to the current interglacial period (Wright et al. 1993). Nevertheless, not all species and populations will have the option of moving. Nonvolant species on mountain tops or other habitat islands will have nowhere to go. Similarly, isolated foci of some vector-borne diseases that rely on the continued presence of certain hosts and vectors could disappear if movement of either is restricted. Although climate change might result in suitable climatic conditions elsewhere, these areas will remain disease-free unless all components of the system (host, vector, pathogen) reach and occur simultaneously in these sites for sufficient time to establish transmission cycles. Anthropogenic habitat fragmentation as a result of deforestation, agriculture, road building, construction of towns and cities, and other land use changes will impede migration, jeopardizing the existence of some populations and species (Root and Schneider 2002). A mathematical model was used to predict that > 400 (relatively mobile) bird species will suffer > 50% range contractions and > 50 species will become extinct because of combined effects of climate change and anthropogenic habitat loss (Jetz et al. 2007). According to the IPCC’s *Fourth Assessment Report*, 20–30% of plant and animal species are likely to be at increasingly high risk of extinction as global mean temperatures exceed 2–3°C above preindustrial levels (Fischlin et al. 2007).

It seems intuitive that extinctions resulting in fewer species of host animals should decrease the number of potential zoonotic pathogens and thus the incidence of VBZDs. Several scientists have suggested that high biodiversity is causally related to high levels of infectious disease, both locally and regionally (Sattenspiel 2000; Wolfe et al. 2005). Indeed, some highly specific pathogens are likely to disappear with their hosts. Nevertheless, extinction and biodiversity loss may have some counterintuitive affects on the incidence of some VBZDs. Extinctions will occur selectively with habitat and dietary specialists being affected first (MacDonald and Brown 1992; Warren et al. 2001). Meanwhile more opportunistic, generalist species may not only survive but thrive in the absence of competition from specialists. In their study of climate change and altitudinal migration in Yosemite National Park, Moritz et al. (2008) found that opportunistic small mammal species (those that were short-lived and had more litters per year) were more likely to expand their range upward than were their long-lived, less fecund counterparts. The host species for most of the known rodent-borne hemorrhagic fever viruses that affect humans are opportunistic species (Mills 2006). Although it is possible that this pattern is due, at least in part, to sampling error (diseases associated with anthropophilic species would be the first to come to our attention), the pattern could be the logical consequence of several characteristics of opportunistic species (high vagility, rapid growth, early reproduction, high fecundity, and capacity to sustain high population densities that are conducive to the transmission of pathogens) that would facilitate the evolution and maintenance of pathogens. Further, decreases in the species diversity of potential host assemblages have been associated with increased prevalence of infection by zoonotic pathogens in host populations both for vector-borne diseases (Ostfeld and Keesing 2000) and for nonvector-borne zoonoses (Mills 2005). Thus, selective extinctions associated with climate change can result in a net increase in the risk of some zoonotic infections in both wildlife and humans.

### Other interactions and confounders

In addition to barriers caused by anthropogenic changes, natural barriers to dispersal such as mountain ranges or bodies of water can preclude species from reaching suitable habitats. Stronger competitors or predators or the absence of prey or other food sources could prevent species from occupying otherwise suitable habitats.

Behavioral adaptation such as changing activity patterns or nesting sites can alter the responses of individuals and populations to climate change. One factor facilitating the northward range expansion of hispid cotton rats has likely been a switch to occupancy of deeper burrows that extend below the frost line (Dunnum 2002).

Species with the genetic capacity to undergo evolutionary adaptation to a changing environment can withstand or even benefit from climate change. The photoperiodic response of the pitcher plant mosquito (*Wyeomyia smithii*) has shifted toward shorter, more southern day lengths in response to an extended growing season, demonstrating an adaptive evolutionary response to global warming in eastern North America (Bradshaw and Holzapfel 2001). The capacity for such adaptation will vary among species, and those that lack adaptive capacity may be forced to migrate in order to survive.

Some investigators believe recent increases in malaria incidence in the highlands of East Africa were associated with a warming trend that began in the 1970s (Pascual et al. 2006; Patz 2002); others have linked the increases to other factors, including drug resistance (Hay et al. 2002a, 2002b; Shanks et al. 2002). However, the reality may involve the interaction of multiple factors.

Climate changes do not satisfactorily explain the temporal and spatial patterns of increases in incidence of tick-borne disease in Europe. Other factors likely include demographic changes, increases in deer abundance, and changes in land use after the end of communist rule in Eastern Europe (reviewed by Gage et al. 2008).

Finally, in this review, we have often used observations of short-term responses of hosts, vectors, and pathogens to climate variability to conjecture about possible responses to long-term climate change. Responses of host, vector, and pathogen populations to temporary—natural or experimental—changes in environmental conditions (wet or dry periods, warm or cold periods) are clues to how populations will respond to long-term climate change, but these inferences must be made cautiously. North American deer mice, an opportunistic species, quickly respond to increasing moisture in the southwestern United States. Their rapid population growth allows them to escape predators and competitors in the short term. However, if such changes are longer term, competitors will respond to the same changing climatic conditions and predators will adapt to the increased density of prey. The new long-term equilibrium is likely to be much different from what was observed during the initial short-term response. Longer term behavioral, social, and genetic adaptation to the new biotic and abiotic environment will ultimately modify outcomes in ways that may not have been predicted from short-term observations. This important issue, and other caveats for predicting climate change effects on disease, are treated in detail in an earlier review (National Research Council 2001).

In summary, host and vector “responses” that are correlated with (or “associated with,” to use our terminology throughout this review) changing climate variables do not prove cause and effect. Potential confounding variables including behavioral changes, interspecific interactions, intrinsic population phenomena, anthropogenic factors, and evolutionary changes should be considered when appropriate.

## What Kinds of Studies Will Be Useful?

The CDC policy on climate change and public health (CDC 2010) sets forth 11 priority public health actions that address research, prediction, training, communication, preparedness, and prevention to prepare the nation and the world for confronting potential health problems associated with climate change. Using these general priorities, we propose six specific goals that we believe would lead to a better understanding of the interaction between climate and VBZDs and an improved capability for predicting how climate change will affect the incidence and distribution of VBZDs.

### Pathogen identification and characterization

Our knowledge of tropical biota and the pathogens associated with them is inadequate [see Supplemental Material (doi:10.1289/ehp.0901389)]. Calls for the study of worldwide (and especially tropical) biodiversity have come from a variety of sources [e.g., the United Nations, the U.S. National Science Foundation (NSF), and the Ecological Society of America]. Understanding biodiversity of plants and animals has scientific, economic, and esthetic appeal and has attracted much support (e.g., NSF’s Planetary Biodiversity Inventories program; NSF 2003). Understanding the diversity of tropical pathogens has obvious practical importance yet has received little attention. Although a complete survey of potential pathogens in nature is an overwhelming task, a useful beginning would be to select the most likely potential hosts and vectors (e.g., bats and rodents, ticks and mosquitoes) and catalog those groups of pathogens with known propensities for causing disease in humans (e.g., hemorrhagic fever viruses, arboviruses, rickettsiae, and some bacteria).

Establish baseline data on the geographic and habitat distribution of recognized zoonotic and vector-borne pathogens and their hosts and vectors

Accurate information on the current distribution of these agents and their hosts and vectors defines current potential disease-endemic areas and helps to quantify relative risk among habitat types. These data also are prerequisite to documenting spatial changes in the distribution of pathogens associated with climate change. Because pathogen distributions do not always coincide with host and vector distributions (Mills and Childs 1998), studies cannot be limited to documenting host and vector presence or absence but must include sampling hosts and vectors for pathogen presence. Finally, because distinct pathogens may be associated with genetically distinct populations of hosts or vectors (e.g., at the subspecies level) (Mills and Childs 1998), accompanying taxonomic studies will be required. Collection of accurate baseline data is the first step in the establishment of long-term monitoring programs.

### Establish longitudinal monitoring programs

A major impediment to accurate prediction has been the lack of long-term monitoring programs (Harvell et al. 2002; Jannett et al. 2007; Pascual and Bouma 2009; Sagarin 2002). These studies would track changes in zoonotic disease risk to humans by following changes in population dynamics and infection prevalence of a few zoonotic pathogens in their host and vector populations. These studies, whose length should be measured in decades, would monitor changes in environmental variables using field-based and remote-sensing platforms and identify factors associated with changes in host, vector, and pathogen dynamics at selected sites. Long-term sampling should also monitor changes in the distribution of hosts and vector populations and their distribution across latitudinal, elevational, and environmental gradients. Although only a few agents, hosts, and vectors can be addressed this way, these studies might be applied to other pathogens, even those yet to be discovered, that have the same or similar host or vector associations.

A few long-term databases describing temporal dynamics of animal populations or disease incidence already exist (e.g., data from long-term ecological research sites and records of reportable diseases), and a few more are proposed (e.g., NSF’s National Ecological Observatory Network), but such ecological data were rarely collected for the purposes of infectious disease studies. Conversely, surveillance records for human diseases rarely contain significant amounts of useful ecological data. Nevertheless, some of these data sets might be paired with long-term weather data to provide insight into climate effects (Pascual et al. 2002; Stenseth et al. 2006).

Track data on the geographic distribution, severity, and frequency of outbreaks of wildlife diseases and VBZDs in humans

This is another type of longitudinal monitoring program, but the list of pathogens and hosts would be broader and the data collected more restricted (e.g., geographic coordinates, dates, number of cases). These data can be used to track geographic and temporal trends in VBZD incidence, identify vulnerable populations, and test predictions based on forecasting models. Several existing databases provide useful formats (e.g., U.S. Geological Service’s Global Wildlife Disease News Map; National Biological Information Infrastructure 2010), the CDC’s ArboNet, and the Global Early Warning System for Major Animal Diseases including Zoonoses [World Health Organization (WHO) 2010]. Integration of such formats with the monitoring, data gathering, and data dissemination capacity of CDC and WHO networks would provide a powerful tool, making geographic and incidence data quickly available to the broad community of public health professionals, researchers, and modelers.

Conduct experimental laboratory and field studies of effects of climate change on hosts and vectors and their abilities to maintain and transmit pathogens

Laboratory studies and manipulative field studies (Post et al. 2008) can be used to test mechanisms of climate change effects on hosts, vectors, and pathogens or to provide hypotheses for field testing. Because specific environmental factors cannot be isolated and controlled in the field, laboratory studies will be the best way to test the effects of specific changes in temperature, humidity, or physiological stress on host, vector, or pathogen populations. For example, stress-related immunosuppression has been suggested as an explanation for increased rates of transmission and for viral recrudescence including increases in pathogen load in hantavirus hosts observed in field studies (Botten et al. 2003; Kuenzi et al. 2005). Laboratory experiments provided preliminary evidence in support of this hypothesis in the case of Seoul hantavirus infection in Norway rats (*Rattus norvegicus*) (Klein et al. 2004). Additional controlled laboratory experiments will be necessary to confirm this hypothesis for other VBZD pathogens and to help quantify the environmental and social factors (temperature, crowding, aggression, breeding, etc.) associated with transmission and recrudescence.

Neither field nor laboratory studies alone can provide complete answers to these questions. Physiological patterns observed in the laboratory must be tested under natural field conditions and the physiological mechanisms for patterns observed in the field must be tested in the laboratory.

Use data from laboratory and field studies, epidemiological studies, and remote sensing to develop predictive models of changes in zoonotic disease risk and the projected distribution and abundance of major hosts and vectors

The utility of such predictive models has been proven. Long-term direct monitoring of host population density and prevalence of hantavirus infection in North American deer mice provided early warning of elevated risk for HPS in the southwestern United States (CDC 1998, 1999; Yates et al. 2002). Models have used rainfall and temperature data to identify high-risk areas for plague and HPS in the same geographic area (Eisen et al. 2007; Enscore et al. 2002; Parmenter et al. 1999). Remote sensors have demonstrated climate-associated changes in vegetation or other environmental parameters that foretold increased risk for Rift Valley fever (Anyamba et al. 2001) or HPS (Glass et al. 2002). Ecological niche modeling has been used to provide evidence for climate change-mediated range shifts for plague and tularemia and to provide predictions of continued future poleward expansion (Nakazawa et al. 2007). Ecological niche models also used climatic data and other environmental datasets to predict the geographic distribution of the hosts and vectors for Chagas disease and the filovirus hemorrhagic fevers (Peterson et al. 2002, 2004). Another model predicted the northward spread of the Lyme disease and anaplasmosis vector *I. scapularis* under proposed climate change scenarios (Brownstein et al. 2005). Recent surveillance has confirmed that expansion (Ogden et al. 2010). Continued data collection from long-term field studies combined with laboratory data providing measured physiological tolerances for specific hosts and vectors and effects of stressors on pathogen transmission can be used to develop increasingly accurate models of range expansions and effects of increased population density on heat stress and pathogen transmission.

Although predictive models may accurately identify the changes in suitable physical habitat for host and vector species, it is important to remember that the realized niche (actual range of conditions occupied by a species) is always smaller than the fundamental niche (potential range in the absence of predators, competitors, and other limiters); the many nonclimatic confounders discussed above (barriers to dispersal, predation, competition) will need to be included in accurate models (Lafferty 2009).

## An Ecosystem Approach

Over the last few decades it has become clear that effective disease prevention must consider the whole environment in which disease occurs. The maintenance of healthy people requires the maintenance of healthy ecosystems. Understanding ecosystem changes in relation to climate change will require not only a clear understanding of future changes in the physical environment but also a precise understanding of the physiological, ecological, and evolutionary responses to these changes by pathogens, vectors, and vertebrate hosts. Because of the complexity of pathogen–vector–host interactions, the multiple interactions involved, and the environment in which these interactions are occurring, studies must be multidisciplinary, long term, spatially diverse, and sufficiently replicated to provide conclusions that are reliable and generalizable. No single institution has the expertise and resources to establish multiple, intensive, long-term field monitoring programs and appropriate laboratory experiments, collect and interpret satellite images, and apply these data to the development of mathematical simulation models and forecasting tools. Collaborative partnerships among government and nongovernmental organizations and universities will have the best chance of achieving these goals. In order for broad collaborations to be most effective, mechanisms must be developed for effective communication, data sharing, and research integration among institutions and disciplines.

## Application to Public Health

An important product of these studies would be an enhanced ability to initiate mitigation strategies, promulgate early warning information, and target response capabilities. Predictions based on long-term monitoring programs and forecasting models should be quickly disseminated to local, state, and international public health partners. In consultation with those partners, intervention strategies should be developed to mitigate the impact of disease on human populations. In response to predictions of increased risk of HPS in the southwestern United States in 1998 and 1999, the CDC, in partnership with several southwestern States, developed and promulgated prevention messages via television, radio, pamphlets and posters. Despite large increases in local rodent host populations, numbers of human cases were lower than in the previous HPS outbreak in 1993/1994 (CDC 1998, 1999).

Prediction and promulgation efforts can be enhanced through integration with several existing U.S. government initiatives directed at climate change, including *a*) Climate Mapper, which makes the results of climate change models accessible to a broad user community; *b*) the Malaria Early Warning System, which recently examined rainfall patterns in sub-Saharan Africa using meteorological station data, rainfall estimated from satellite images, and malaria incidence; and *c*) the Global Earth Observation System of Systems, which is an ambitious undertaking to coordinate disparate earth-observation systems across the world including improved sharing of remote-sensing data.

## Conclusions

Reliable data on the effects of climate change on VBZDs are scant. We have summarized the data from preliminary studies and used these data to extrapolate to logical conclusions concerning potential consequences of climate change on VBZDs. Nevertheless, these conclusions will remain speculative until tested by rigorous field and laboratory studies. We emphasize the importance of such studies and propose a series of goals that we believe would lead to improvements in our understanding of, and our ability to mitigate, the effects of climate change on VBZDs.

The goals of the actions outlined above are to form the multidisciplinary relationships that are need to conduct and interpret ecosystem-based studies of VBZD pathogens in host and vector populations; identify the hosts, vectors, and pathogens (likely to be largely tropical) with the greatest potential to affect human populations under climate change scenarios; and conduct studies that will increase our understanding of the four potential mechanisms by which climate change may affect VBZD incidence. The value of these studies is not limited to the context of climate change. We believe that most, if not all, of these goals are important in their own right and should be undertaken regardless of the additional urgency added by the unknown effects of climate change. The goals we propose are broad and general. For each of these goals, a research plan with specific objectives, methods, and deliverables should be developed. Such research plans will be most effectively conceived in a multidisciplinary environment where experts in ecology, wildlife and vector biology, and public health sit together at the planning table. The requirements for multiinstitutional and multidisciplinary studies are important. Understanding and mitigating the effects of VBZDs cannot be accomplished by the public health community without the collaboration of ecologists and wildlife biologists.

The primary focus of our proposals is discovering and predicting the consequences of climate change on VBZDs and driving public health action for the development of more effective disease-prevention strategies. We are not the first to suggest research goals to address the understanding and mitigation of climate change effects on VBZDs and other infectious diseases. However, for many aspects there is a clear convergence of conclusions and research recommendations (see, for example, National Research Council 2001; Semenza and Menne 2009). The sooner these studies are undertaken, the sooner intervention strategies can be developed and instituted.

## Figures and Tables

**Table 1:** Some of the important VBZDs mentioned in this review, with their etiological agents, vectors, and vertebrate hosts.

Disease	Pathogen	Vector	Vertebrate host
Vector-borne zoonotic diseases			
Lyme disease	*Borrelia burgdorferi* (bacterium)	*Ixodes* spp. (tick)	White-footed deer mouse (*Peromyscus leucopus*) and eastern chipmunk (*Tamias striatus*)^a^
Tick-borne encephalitis (TBE)	TBE virus	*Ixodes* spp. (tick)	Rodents
Rift Valley fever	Rift Valley fever virus	*Aedes* and *Culex* spp. (mosquito)	Livestock^b^
Plague	*Yersinia pestis* (bacterium)	*Xenopsylla cheopis* (flea)	Rodents
Tularemia	*Francisella tularensis*	Ticks (several species)^c^	Rodents, lagomorphs^c^
West Nile fever	West Nile virus	*Culex* spp. (mosquito)	Birds, especially crows and jays
Leishmaniasis	*Leishmania* spp. (protozoan)	*Phlebotomus* and *Lutzomyia* spp. (sand fly)	Many mammal species
Chagas disease	*Trypanosoma cruzi* (parasite)	Triatomine bugs	Many wild and domestic mammals

Vector-borne nonzoonotic diseases			
Malaria	*Plasmodium* spp. (protozoan)	*Anopheles* spp. (mosquito)	Humans
Dengue fever	Dengue virus	*Aedes aegypti*	Humans

Nonvector-borne zoonotic diseases			
Hantavirus pulmonary syndrome (HPS)	Sin Nombre virus	None	North American deer mouse (*Peromyscus maniculatus*)
	New York virus	None	White-footed deer mouse (*Peromyscus leucopus*)
	Black Creek Canal virus	None	Hispid cotton rat (*Sigmodon hispidus*)
	Numerous viruses in South and Central America	None	Rodents of subfamily Sigmodontinae
Hemorrhagic fever with renal syndrome	Seoul virus (and other Eurasian hantaviruses)	None	Norway rat (*Rattus norvegicus*; for Seoul virus) and several other rodents of subfamily Murinae
South American hemorrhagic fevers	Arenaviruses	None	Rodents of subfamily Sigmodontinae
Monkeypox	Monkeypox virus	None	Terrestrial rodent species
Filovirus hemorrhagic fevers	Marburg virus and Ebola virus	None	Egyptian fruit bat (*Rousettus aegyptiacus*) and likely other bat species
Nipah virus encephalitis	Nipah virus	None	Fruit bats (*Pteropus* spp.)

a Principal hosts in eastern North America; other rodent species are hosts in western North America, Europe, and Asia.

b Virus survives in eggs of infected mosquitoes; vertebrate reservoir not required for viral maintenance.

c Vectors, hosts, and routes of transmission are highly variable.
